# Coating Materials to Prevent Screw Loosening in Single Dental Implant Crowns: A Systematic Review

**DOI:** 10.3390/ma17205053

**Published:** 2024-10-16

**Authors:** Lara Coelho, Maria-Cristina Manzanares-Céspedes, Joana Mendes, Victòria Tallón-Walton, Wilson Astudillo-Rozas, Carlos Aroso, José Manuel Mendes

**Affiliations:** 1 UNIPRO—Oral Pathology and Rehabilitation Research Unit, University Institute of Health Sciences (IUCS), CESPU, 4585-116 Gandra, Portugal; lara.coelho@iucs.cespu.pt (L.C.); joana.silva.mendes@iucs.cespu.pt (J.M.); jose.mendes@iucs.cespu.pt (J.M.M.); 2 Human Anatomy and Embryology Unit, Faculty of Medicine and Health Sciences, University of Barcelona, 08007 Barcelona, Spain; vtallon@ub.edu (V.T.-W.);

**Keywords:** chlorhexidine, dental implants, dental prosthesis implant supported, friction, torque, lubricants, mechanical complications, preload, removal torque value, silicones

## Abstract

Oral rehabilitation with dental implants has resulted in high success rates. However, some complications have been described, such as the loss of the prosthetic screw. Some manufacturers sell screws with different coatings to avoid screw loosening, but even these types of screws can come loose. We aimed to investigate the screw coatings that can be applied during a dental appointment to avoid screw loosening. Following PRISMA Guidelines, we searched PubMed/Medline, Embase and Web of Science for studies published up to January 2024. All studies of single dental implant crowns, in which the prosthetic screw was coated with a lubricant and the preload and/or the removal torque value (RTV) was recorded, were analyzed. We excluded studies applying the finite element method (FEM) as well as studies without a control group. The risk of bias was assessed with a tool developed by our research group. Of the 1959 records identified, 19 were selected. Ten studies were considered to have a low risk of bias, and nine were considered to have a medium risk of bias. The coatings tested were adhesives, saliva, chlorhexidine, Vaseline, silicone gel, Polytetrafluoroethylene (PTFE) tape, blood, fluoride, Listerine^®^ Mouthwash and normal saline. The preload, the RTV with and without cyclic loading and the percentage of RTV loss were recorded. Some coatings show promise, although there is no clear evidence that any option is superior in minimizing screw loosening.

## 1. Introduction

Partially edentulous patients currently represent the main group of candidates for oral rehabilitation with implants; in the long term within this group, there have been good survival rates for implants. However, some biological and mechanical complications have been reported, such as the loss of the prosthetic screw, which is the most cited complication with a prevalence between 4.6 and 12.7% [1,2,3]. Several strategies have been developed to reduce the incidence of screw loss, which are related to (1) prosthesis design (implant well positioned, occlusal contacts as centered as possible, mutually protected occlusion and reduction of the cantilever and cusp inclination, as well as occlusal light contacts) [1,4,5,6]; (2) screwing/fixation techniques (re-screwing the screw 10 min after the first screwing, increasing the torque above that recommended by the manufacturer, techniques to ensure correct screw engagement and coating of the screws with coating materials) [7,8,9,10]; and (3) screw/implant properties (related to screw design such as size or head, material and brand of the screw itself, use of screws with the treated surface, design of the internal thread of the implant and the type of connection of the implant using two screws) [7,8,11,12,13,14].

As far as unscrewing is concerned, it is essential to understand the mechanics of the screw. The prosthetic screw is intended to functionally join two components: implant and prosthetic abutment. Thus, the abutment is screwed by applying a torque that creates a series of forces in the abutment–implant complex called preload. When the screw is tightened, it elongates, producing tension. Its elastic recovery tightens the two components of the prosthesis, creating a clamping force [15]. Of the applied torque during clamping, 50% is spent on overcoming the friction between the screw head and the seating surface of the prosthetic abutment. Approximately 40% of the applied torque is applied to overcome lap friction and 10% is applied to tighten the screw [14]. In addition, the settling effect plays a critical role in the stability of the screw; it is the result of the screw surface not being completely smooth, and because of this microroughness, the screw and implant are not completely in contact. Settling occurs when the rough spots flatten out. Studies [4,15,16] report that around 2–10% of initial preload is lost due to the settling effect, which is directly related to the surface roughness and hardness of the surface, as well as the magnitude of the applied torque. Thread friction is higher in the first adjustment and decreases with following repetitions. In this way, it has been verified that the torque required to untighten the prosthetic screw—removal torque value (RTV)—is less than the torque initially used to tighten it [4,15,16].

The amount of preload present at the threads of the prosthetic screw depends on the (1) applied torque; (2) presence of materials or substances that act as lubricants; (3) physical properties of materials; and (4) settling effect [17]. Silva-Neto JP et al. found that preload is highly important in preventing screw loosening [18]. Several studies have evaluated ways to increase the preload without increasing the torque, based on the coefficient of friction reduction using various coating materials already applied on the screw and sold by the manufacturers, such as pure gold, tungsten carbide and diamond-like carbon [16,19,20]. Even so, some screws come loose after placement and function, which generates the need to evaluate the coating materials that could be applied during a dental appointment. Some compounds such as saliva (human and artificial), blood, Vaseline, chlorhexidine (CHX) and fluoride have also been extensively studied for their lubricating action [21]. The application of a sealing gel, a polytetrafluoroethylene (PTFE) tape and adhesives have also been evaluated to solve the loss of the prosthetic screw [7,9,22,23].

The aim of this systematic review was to investigate the current literature on the different screw coating materials that can be applied during a dental appointment to prevent abutment screw loosening in single-tooth implant crowns and analyze their influence on the preload and RTV.

## 2. Materials and Methods

The present review was conducted according to the Preferred Reporting Items for Systematic Reviews and Meta-Analyses (PRISMA 2020) Guidelines [24] but was not registered in the International Prospective Register of Systematic Reviews (PROSPERO) because of the in vitro nature of the studies. The PRISMA checklist is provided in the Supplementary Materials. The procedures and methodology are available online (https://osf.io/cz4df, accessed on 11 October 2024). The proposed focus question was as follows: Can the application of lubricant on the abutment screw in single crowns on dental implants reduce the likelihood of abutment screw loss?

*Eligibility Criteria:* The eligibility criteria were formulated according to the acronym PICO: (Population) patients with single dental implant crowns; (Intervention) application of a coating; (Comparison) screw-retained abutments with and without coatings; and (Outcomes) the preload and RTV. The inclusion criteria were as follows: (1) studies using dental implants with single crowns; (2) studies with a control group without any coating and with at least one test group with a coating application; (3) studies analyzing at least the RTV or percentage torque loss calculated from the RTV; and (4) studies in which the coating could be applied in one appointment with the dentist. The exclusion criteria were as follows: (1) studies whose results were obtained by finite element methods (FEM); (2) studies without a control group; and (3) studies with missing data.

*Information Sources and Literature Search Strategy:* An individual search strategy was developed for each of the following electronic databases: PubMed/Medline, Embase and Web of Science. The searches were conducted in January 2024. No time or language restrictions were applied. Medline articles were retrieved using several MeSH terms, such as “Dental implant”, “Dental Prosthesis Implant Supported”, “Dental Abutment”, “Torque”, “Friction”, “Dental Restoration failure”, “Lubricants”, “Lubrication”, “Chlorhexidine”, “Silicones”, “Adhesives” and “Coated materials, biocompatible”. Similar sensitive search strategies were adapted for the Embase, the use of Emtrees, and the Web of Science database. All the MeSH terms and Emtrees were combined with “OR” using natural language.

*Study selection:* After the removal of duplicates using Excel (Microsoft Office^®^ 2021, MSFT, Software para Microcomputadores, Lisboa, Portugal), the review process consisted of two phases: During the first phase, two reviewers independently examined the titles and abstracts of the records identified through the electronic databases (authors L.C. and M.C.M.). Any disagreement was resolved by discussion, and in case of doubt, the full text of the article was obtained. In the second phase, two independent reviewers retrieved the full texts of possibly relevant studies for a final analysis (inclusion or exclusion in the review). Disagreements were resolved by consensus or, if they persisted, by the decision of a third independent reviewer (author J.M.M.). When necessary, the study authors were contacted to obtain additional information. When the final selection was made, all studies were exported to Zootero^®^ (version 7.0, Center for History and New Media, George Mason University, Fairfax, Virginia, USA).

*Data Extraction and Items:* The same two reviewers (authors L.C. and M.C.M.) independently extracted the data of interest using Excel (Microsoft Office^®^ 2021) and constructed a table with the author(s) and year of publication, the implant connection, the samples and coatings, the screw material, the tightening torque, the coating material tested, the test protocol, the cyclic loading characteristics and the main outcomes. Any disagreement in data extraction was resolved by consensus, and when it persisted, the third reviewer was consulted.

*Quality Assessment:* To establish the quality of the included studies, two reviewers (authors L.C. and M.C.M.) developed risk-of-bias assessment criteria for in vitro studies (adapted from Samuel et al. [25]). They considered 12 parameters divided into 4 groups: Appropriate Statistical Methods, Execution, Analysis of Bias and Presentation of Results. The defined parameters were whether (1) a sample size calculation was performed, (2) an appropriate statistical analysis was performed, (3) there was an appropriate control group, (4) the tests were conducted according to a standardized protocol (such as ISO or American Society for Testing and Materials (ASTM)), (5) the test substance was defined, (6) the characteristic description of the study design was described, (7) there was blinding of the investigators, (8) the experiment was performed by a single operator, (9) the conflict of interest statement/funding was recorded, (10) the authors had accounted for confounding variables, (11) there were any incomplete outcome data and (12) there was a selective reporting of results. Each parameter received a score of 0 if it was clearly reported, 1 if it was reported but was inadequate or unclear and 2 if the information could not be found. Studies that received scores of 0 to 8 were classified as having a low risk of bias, a score of 9 to 16 was considered medium risk and a score of 17 to 24 was considered high risk. The two authors independently applied the tool to each included study and recorded supporting information and justifications for the risk-of-bias judgments for each domain. Any discrepancies were resolved by discussion to reach a consensus, otherwise the third author acted as a referee.

*Effect Measures:* We planned to assess the effect of the abutment screw coating, analyzing the screw preload and the RTV outcomes by calculating the risk ratio (RR) of a successful outcome. Because the included studies used different measurement scales, we used standardized mean difference (SMD), effect sizes (Cohen’s) and their 95% confidence intervals.

## 3. Results

### Study Selection

**Figure 1:**
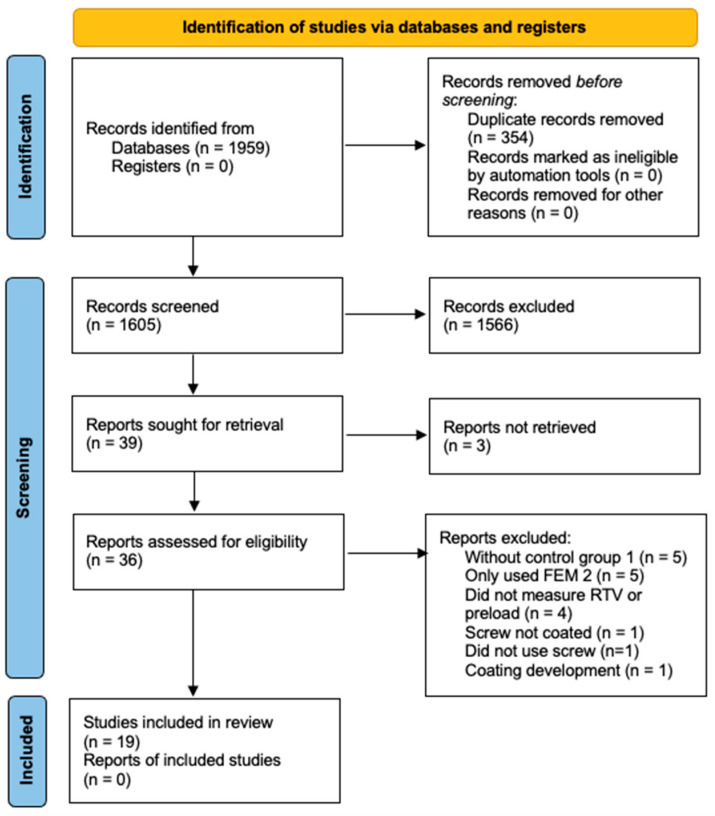
Flowchart of the screening process (PRISMA format).

After performing the electronic search, a total of 1959 studies were identified (870 in PubMed, 326 in Embase and 763 in Web of Science). After removing the duplicates (354 studies), 1605 studies were selected. An initial screening of titles and abstracts resulted in 39 studies that potentially met the eligibility criteria. The search strategy is illustrated by a PRISMA flowchart (Figure 1). A total of 19 of the remaining 37 studies selected for full-text analysis met the selection criteria and were considered eligible for the review.

Characteristics of the Included Studies: A total of 19 in vitro studies were selected for data extraction, and their main characteristics are summarized in Table 1.

Table 2 shows the risk of bias of the included studies. Ten studies [7,23,26,28,30,32,33,38,39,41] were considered to have a low risk of bias, and nine [22,27,29,31,34,35,36,37,40] were judged to have a medium risk of bias.

The studies were published from 2010 to 2023. Six studies were performed on implants with external connections [7,23,28,34,36,38], five were on implants with internal connections [27,29,30,31,39], five were on implants with an internal with a conical connection [26,33,35,40,41], one used implants with an internal connection and an internal with a conical connection [22], one used external and internal connections [32] and one used analogs and did not report the connection [37].

The samples used in each group were as follows: 10 studies used 10 samples [23,26,28,29,30,32,33,34,36,37], 2 used 15 samples [7,40], 1 used 7 samples [35], 2 used 5 samples [22,27], 1 used 12 samples [38], 1 used 11 samples [41], 1 used 9 samples [31] and 1 used 8 samples in the CG and 7 in the TG [39].

The studies included different screw materials, namely TorqTite^®^ [34], titanium grade IV [7,30,33,41], titanium (with no grade report) [28,36] and Ti-6Al-4V alloy [32], and eleven studies did not report the screw material [22,23,26,27,29,31,35,37,38,39,40].

The tightening torque was according to the manufacturers’ recommendations, ranging between 20 and 35 N cm; only one study did not report the tightening torque [35].

Several coating materials were tested: bio-adhesive (Bis-GMA + TEGDMA + MMA), saliva (human and artificial), CHX gel 0,2%, Vaseline, silicone gel (KieroSeal^®^ and GapSeal^®^), CHX gel 1% and 2%, CHX Mouthwash 0,2% and 0,1%, PTFE tape, blood, fluoride mouthwash 0.2% and 0.025%, Loctite^®^ (277, 243, 242 and 2400), normal saline, ASMT, ASHT, CYAB and Listerine^®^ Mouthwash.

Regarding the tests, there were differences: six authors did not submit the samples to a cyclic loading machine [23,27,29,34,36,37], three of them only measured the RTV after tightening the screw [34,36,37], two measured the screw preload and the RTV [23,29] and one measured the screw preload, the RTV and percentage torque loss [27]. Two authors only carried out thermocycling and evaluated the RTV after that [30,31], two authors carried out thermocycling and cyclic loading and evaluated the RTV after that [33,41], eight authors carried out cyclic loading [7,22,26,28,32,38,39,40] and one carried out dynamic cyclic loading [35]. Some authors [7,26] also evaluated the RTV after cyclic loading, while others [28,39,40] evaluated the RTV before and after cyclic loading. Lyra et al. [32] and Ozdiler et al. [35] evaluated the percentage torque loss after cyclic/dynamic loading, Seloto et al. [38] evaluated the screw preload and the RTV after cyclic loading and Yu et al. [22] evaluated the percentage torque loss before and after cyclic loading.

All authors who carried out thermocycling [30,31,33,41] used the same test protocol: 1500 cycles, temperature between 5 and 55 °C and 60 s of dwell time. The authors who performed the cyclic loading differed in the number of cycles of the protocol test, which varied between 300,000 and 1,200,000, and the applied load was either specific (varying between 50 and 200 N) or dynamic (0–200 N, 20–200 N and 11–211 N).

The frequency was also different, varying between 1 and 15 Hz.

The sample angulation was not used by all authors, but those who used it used a 30° off-axis angulation [7,22,28,38,39].

In studies that were not submitted to cyclic loading, one [29] found no significant results testing KieroSeal^®^, CHX and Vaseline. However, Asli et al. [27] stated that the CHX gel significantly decreased the RTV, but others [23,34,36,37] found that coating with saliva, an adhesive, blood and normal saline, a sealing agent for high torque and a cyanoacrylate-based bond significantly increased the RTV.

Some authors [7,26,33,41] found that the application of an adhesive, PTFE tape, fluoride mouthwash, CHX mouthwash and blood significantly raised the RTV after cyclic loading.

Of the authors who only measured the RTV after thermocycling, Gumus et al. [30] found that blood reduces the RTV significantly and Koosha et al. [31] found that the CHX mouthwash raised the RTV significantly while human saliva decreased the RTV significantly.

When analyzing the percentage torque loss, two authors [27,35] found a significant increase by applying CHX gel and KieroSeal^®^, and two authors [22,32] found a significant decrease by applying an adhesive and GapSeal^®^.

Two authors [38,40] found that the application of an anaerobic sealing gel and Listerine^®^ Mouthwash increased the RTV after cyclic loading. On the other hand, Shemtov et al. [39] found that coating the screws with CHX mouthwash, CHX gel and fluoride decreased the RTV after cyclic loading. Basilio et al. [28] tested the application of Vaseline and submitted it to cyclic loading but found no significant differences.

## 4. Discussion

The objective of the systematic review was to investigate whether there is a screw coating material that can be applied during a dentist’s appointment in order to maintain the screw and prevent it from loosening again. Considering that the list of coatings used is extensive and with contradictory results, we have organized the discussion by type of coating.

Adhesives: An adhesive is expected to provide a strong adhesion between the screw and the implant, sealing the micro gaps and lubricating the screw, thus increasing the preload. All studies found significant differences in most of the tested adhesives when coating the screws; they found a higher RTV [23,26,36,38] and a reduced percentage of torque loss after cyclic loading [32]. The only unexpected result was for the ASMT, which did not show significant results [23]. These results can be attributed to the adhesive’s wetting properties, its strong adhesiveness and its ability to overcome friction forces [26]. It should be noted that some of the adhesives tested had an RTV higher than the installation torque; in fact, in one study the authors reported screwdriver fractures during de-torque [23], which could contraindicate an adhesive’s clinical use, as sealing agents should allow loosening of the screw when necessary. Another limitation of this procedure is that the anaerobic sealing gel takes between 24 and 72 h to complete the polymerization [23,26,36,38]; therefore, the RTV measurements were only carried out after this time, which makes it impossible to transfer this to reality, as it is not usual for a crown to be without function during this time. Finally, excepting one study [26], none of the authors tested the biocompatibility and cytotoxicity of the adhesives, so for now, although adhesives achieved good results in preventing screw loosening, their use in vivo should not be recommended.

Sealing Silicone Gel: The application of a sealing gel, which is commonly used in implantology to protect the implant against bacterial colonization, thus preventing peri-implantitis, was tested as a screw coating. Theoretically, it could decrease the coefficient of friction on the screw and consequently result in an increase in the preload force; therefore, it is hypothesized that the high viscosity of the gel could act as a protective layer, helping to cushion the external loading impact to preload and preventing de-torque [22]. One of the studies [29] applied KieroSeal^®^, without performing cyclic loads; they found that its application did not influence the screw preload. However, when analyzing the RTV, they found that in the Nobel^®^ implant group, KieroSeal^®^ had the highest RTV, while in the Bego^®^ implant group, the CG had a higher RTV than the KieroSeal^®^ group. In this study, the authors did not assess whether there was a statistically significant difference between groups, and we cannot draw many conclusions about the effect of this sealing gel. In the other two studies, both performed cyclic loads. Ozdiler et al. [35] found that the application of KieroSeal^®^ significantly increased the percentage torque loss, while Yu et al. [22] found that GapSeal^®^ significantly decreased the percentage torque loss. This result can be justified by the difference in the cyclic loading definitions: Yu et al. [22] followed the ISO 14801 [42] standards by angulating the samples at 30° off the axis and Ozdiler et al. [35] performed a full metal crown restoration with 30° cusp inclinations and cemented with a resin cement and carried out a dynamic loading. The number of cycles were also different: Ozdiler et al. [35] performed 500,000 cycles, while Yu et al. [22] performed 345,600. Another important difference consisted of the sealing gel itself, which had a different composition. Ozdiler et al. [35] stated that this result may be related to the setting shrinkage behavior of the silicone materials, as they had an initial dimensional change due to polymerization. Additionally, there was the possibility that a thin layer of sealant gel remained at the junction between the implant and abutment, and when retightened 10 min later, it had already hardened, thus naturally interfering with obtaining the desired torque. Yu et al. [22] used GapSeal^®^, which, according to its characteristics, is a highly viscous material that never hardens, thus preventing shrinkage gaps. Yu et al. [22] evaluated the initial and final torque loss rate, and the results in the group sealed with GapSeal^®^ were significantly better, indicating that its application improved preload.

PTFE Tape: The only study [7] evaluating the effects of applying PTFE tape to the screw showed a significantly higher RTV after cyclic loading. It is impossible to compare results, as we did not find any more studies that applied PTFE tape. The authors stated that the use of PTFE tape could reduce the loss of preload and maintain the initial force applied to the abutment screws.

Saliva: It was hypothesized that saliva debris may infiltrate micro gaps, introducing microorganisms and proteoglycans at the implant–abutment interface. These substances could act as lubricants, with viscoelastic properties that distribute loads and reduce friction [31]. Several studies evaluated saliva. Some studies [27,30,33,37,39,40,41] found that it had no impact on the preload, RTV or percentage torque loss, as the results were not statistically significant, despite the presence or absence of cyclic loading. However, Koosha et al. [31] used human saliva and found that it significantly reduced the RTV after cyclic loadings, while Nigro et al. [34] used artificial saliva and found that it significantly increased the RTV. This result might be due to the saliva tested (e.g., artificial saliva is a solution without protein, glycoprotein sugar or amylase macromolecules), the absence of cyclic loadings and the screw used, which was the TorqTite^®^ (a Teflon-coated titanium screw) instead of a grade IV titanium screw. Studies show that these coated screws reduce the coefficient of friction [16,43], increasing the preload of the screw.

Vaseline: Some authors state [16,43] that the use of Vaseline reduces the friction coefficient, allowing a higher preload to be obtained; however, we did not find the same. Two studies [28,29] evaluating the influence of the application of Vaseline to the screw verified that it had no influence on the preload or RTV, as none of the results obtained significant differences.

CHX: The application of CHX, either in gel or liquid form, is one of the most studied coatings. The ideal screw tightening and preload of the screw are achieved when 60–70% of the yield strength is reached. Reducing the coefficient of friction allows for a higher percentage of yield strength [27]. However, the results are contradictory, as the methodology used in each study is different. For a better understanding and comparison of results, we analyzed the results of applying CHX in both gel and liquid form. Four studies evaluated CHX in gel form, albeit at different concentrations; two did not obtain significant differences [29,35], one found a significantly higher percentage torque loss [27] and the other found a significant RTV reduction only after cyclic loading [27,39]. Asli et al. [27] and Biscoping et al. [29] did not subject the samples to cyclic loading, and the former obtained a significantly higher percentage torque loss, while the latter did not show a significant difference. Asli et al. [27] stated that the observed results were due to the excessive effect of CHX gel in decreasing the friction coefficient; however, the methodology used in this study differs greatly from all others. They used consecutive retightening, a very small sample (only 5), which is unrepresentative, and the percentage of CHX in the gel was the lowest of all. Ozdiler et al. [35] did not find significant differences; also, the methodology used was different. They performed a dynamic cyclic loading (different from ISO14801 [42]) and used implants with four different conical connections. They stated that due to the friction between the abutment and the implant in internal conical connections, the screw is believed to have a relatively minor role in maintaining the connection’s stability. Five studies evaluated CHX in liquid form, all of which subjected the samples to thermocycling or thermocycling and cyclic load. Gumus et al. [30] used the lowest CHX concentration, evaluated the RTV after thermocycling and found no significant difference. Three studies found that the RTV after cyclic loading was significantly higher, which reinforces the idea that some coatings can increase the preload by reducing the coefficient of friction [31,33,41]. Only one study [39] found a significant reduction in the RTV after cyclic loading; they explained that the results were due to the different viscosities of the fluids. When a viscous fluid exists between two surfaces in relative motion, it generates a drag force that opposes this motion and that is increased with the fluid’s viscosity. During screw tightening, the viscosity of the medium introduces additional drag, raising the torque needed to rotate the surfaces and achieve the preload. The viscosities of various media are as follows: blood 3.5–5.5 cP, saliva 2.5–6 cP, fluoride mouthwash > 500 cP, chlorhexidine mouthwash > 100 cP and gel > 200 cP. Variations in the form (gel and liquid) and concentration were assigned different results in previous studies. Kozlovsky et al. [44] reported that the adhesion of CHX to Ti was influenced by the Ti surface roughness and CHX concentration.

Blood: Some authors [31,39] found that contamination with blood does not influence the RTV, and others [33,37,41] found that blood contamination significantly increases the RTV. Only Gumus et al. [30] found a significant reduction in the RTV; they suggested that the accumulation of a biofilm on the abutment screw surface may have a negative effect on the RTV. However, they applied venous blood, which has a higher viscosity and different composition compared with the capillary blood used in the other studies, and it has been shown that blood viscosity can significantly affect the reduction in the RTV after contamination. Consequently, the source of the blood and the time delay when fastening the abutments in the device screw holes may affect the viscosity, leading to variable results in studies. After immediate contact with blood, the interaction between the metal surfaces begins when platelets are attached to the metal surface [33].

Fluoride: Two authors [33,41] performed thermocycling and cyclic loading and verified an increase in the RTV. They explained that a corrosion layer is formed due to an electrochemical reaction in the implant–abutment connection caused by the presence of fluoride. Koosha et al. [31] only performed thermocycling and found no influence on the RTV. Shemtov et al. [39] analyzed the RTV before cyclic loading and also found no influence, but after cycling, they found a significant reduction in the RTV; however, in this study, they used a fluoride mouthwash with the lowest concentration of 0.025%. It seems that the screw material may be important to explain some results. Mostafavi et al. [33] and Yang et al. [41] used titanium grade IV screws, Koosha et al. [31] only reported using titanium grade IV implants, which are resistant to galvanic current and corrosion, and Shemtov et al. [39] did not report the screw material but used Ti-6Al-4V (titanium alloy) dental implants. How the action of fluoride works on the screws is not well explained. Yang et al. [41] claim that the higher RTV was due to the lubricating characteristic of the fluoride feature, while Mostafavi et al. [33] claim that the creation of a corrosion layer explains the higher RTV.

Normal Saline: Only one study [37] tested the influence of normal saline on the RTV and found a significantly higher RTV, although they did not perform cyclic loading. The authors stated that the saline solution was a mixture of sodium chloride in water. The saline solution upon drying had crystalloid particles of sodium chloride that affected the RTV of the abutment screws, but they gave no explanation of how this happened, and we found no other studies testing this coating, so we do not have anything to compare.

Listerine^®^ Mouthwash: Only one study [40] tested the influence of this specific Mouthwash on the RTV before and after cyclic loading and found a significantly higher RTV. The authors also tested artificial saliva and found a significantly higher RTV, although the results of the mouthwash group were better. They explained that the wet medium helps minimize most of the shear forces within the thread and lowers the friction coefficient on its surface, thereby reducing energy loss from friction and allowing more of the tightening torque to be converted into preload.

Limitations: We identified some limitations in this study. Although we found several studies analyzing coatings, some of them were tested only once. Some articles also have very small samples, and as we can see in the risk-of-bias analysis table, most of the articles did not use any rationale in selecting the sample size. In addition, the lack of methodological uniformity is visible; the magnitude, frequency and number of loads reported also vary widely, varying the number of cycles from 300,000 to 1,200,000, the force from 50 to 211 N and the frequency from 1 to 15 Hz. Not all authors followed the most recommended standard for fatigue testing of implants—ISO 14801 [42]. Some claimed to follow it but did not fulfill the standard in its entirety. Another important factor is related to the type of implant connection studied, which is one of the most frequently analyzed factors in previous studies [45]. Many authors reported that screw loosening occurs more frequently in the external connection than in the internal connection and conical connection, as the screw seems to play an insignificant role in maintaining the stability of the connection [35]. It would therefore be more interesting if studies were carried out on implants with external connections. Finally, some studies did not age the samples with cyclic loading, which would replicate the intraoral environment better.

## 5. Conclusions

Based on the findings of this systematic review, the following conclusions can be drawn:

- Adhesives have shown good results in preventing the loss of the prosthetic screw, but their use is not recommended because of the lack of biocompatibility and the difficulty in removing the screw if needed.
- The use of silicone gel sealing has contradictory results; only GapSeal^®^ has promising results, but only one study has evaluated it, so more studies are needed.
- For PTFE tape, Listerine^®^ Mouthwash and normal saline, only one article evaluated these coatings, obtaining promising results, so we must wait for more studies.
- Most articles concluded that contamination with saliva or Vaseline does not seem to have an influence on screw loosening.
- The application of CHX only in liquid form presents favorable results to avoid screw loss, preferably with a concentration of 0.2%; however, the results are not unanimous.
- Coating with blood gives good results if capillary blood is used.
- Coating with fluoride shows good results if the screw used is made of titanium alloy.

Some coatings show promising results, although there is no clear evidence to show that any of the analyzed options are more effective in minimizing screw loosening. However, since reports of the application of GapSeal^®^, PTFE tape, Listerine^®^ Mouthwash, normal saline and capillary blood did not show any results that would contraindicate their use, we suggest that further studies be conducted with these coatings.

## Figures and Tables

**Table 1:** Studies selected for data extraction.

Author, Year	Implant Connection	Sample/Coatings	Screw Material	Tightening Torque	Coating Material Tested	Tests	Cyclic Loading	Outcomes
Arshad et al. (2021) [26]	Internal with conical connection	*n* = 20 Control Group (CG) (*n* = 10): No coating Test Group (TG) (*n* = 10): Adhesive	Not reported (NR)	30 N cm	Bio-adhesive (Bis-GMA + TEGDMA + MMA)	RTV after cyclic loading	Cyclic loading Cycles: 500,000 Load: 75 N Frequency: 2 Hz	Mean RTV value: CG: 20.80 N cm TG: 26.60 N cm With significant difference
Asli et al. (2017) [27]	Internal connection	*n* = 15 CG (*n* = 5): No coating G1 (*n* = 5): Saliva G2 (*n* = 5): CHX	NR	24 N cm	Human Saliva CHX gel 0.2%	Screw Preload RTV and % torque loss	Not present (NP)	Mean screw preload: CG: 24.58 N cm G1 25.08 N cm G2: 24.6 N cm Mean RTV: CG: 19.9 N cm G1: 21.6 N cm G2: 17.28 N cm % of torque loss: CG: 19% G1: 14% G2: 30% No significant difference between the mean screw preload and RTV. However, the % of torque loss was significantly different among all groups.
Basilio et al. (2017) [28]	External connection	*n* = 40 Two types of zirconia abutment: G1 and G2 (*n* = 20 × 2) G1 (*n* = 10): No coating G1 (*n* = 10): Vaseline G2 (*n* = 10): No coating G2 (*n* = 10): Vaseline	Titanium	20 N cm	Vaseline	RTV before cyclic loading RTV after cyclic loading	Cyclic loading Cycles: 500,000 Load: 11–211 N Frequency: 15 Hz Sample angulation: 30° off-axis	Mean RTV before cyclic loading: G1 No coating: 18.5 N cm G1 Vaseline: 18.2 N cm G2 No coating: 15.66 N cm G2 Vaseline: 15.5 N cm Mean RTV after cyclic loading: G1 No coating: 15.4 N cm G1 Vaseline: 15 N cm G2 No coating: 11 N cm G2 Vaseline: 11.4 N cm With no significant difference.
Biscoping et al. (2018) [29]	Internal connection	*n* = 80 2 groups with 2 different implant brands: Bego^®^ (*n* = 40) and Nobel^®^ (*n* = 40) Each group was divided into 4 subgroups: GC (*n* = 10): No coating G1 (*n* = 10): Silicone gel G2 (*n* = 10): CHX G3 (*n* = 10): Vaseline	NR	30 N cm for Bego^®^ implants 35 N cm to Nobel^®^ Implants	Silicone gel (KieroSeal^®^) CHX gel 1% Vaseline	Screw Preload RTV	NP	Mean tightening torque for Nobel Group: GC: 35.04 N cm G1: 35.02 N cm G2 35.07 N cm G3: without results because all the screws fractured when tighten Mean tightening torque for Bego^®^ Group: GC: 30.07 N cm G1: 30.4 N cm G2: 30.07 N cm G3: 30.06 N cm Mean RTV for Nobel^®^ group: GC: 27.44 N cm G1: 26.85 N cm G2: 29.37 N cm G3: NP Mean RTV for Bego^®^ group: GC: 28.47 N cm G1: 29.43 N cm G2: 27.45 N cm G3: 25.5 N cm The authors did not perform a statistical analysis.
Felix et al. (2021) [7]	External connection	*n* = 30 CG (*n* = 15): No coating TG (*n* = 15): PTFE They used the same screws to rescrew and repeat the tests: rCG and rTG	Titanium Grade IV	30 N cm	PTFE Tape (100 micras)	RTV after cyclic loading	Cyclic loading Cycles: 300,000 Load: 200 N Frequency: 2 Hz Sample angulation: 30° off-axis	Mean RTV: CG: 14.46 N cm TG: 19.97 N cm rCG 14.42 N cm rTG: 19.13 N cm With significant differences between the RTVs for the CG and rCG versus rTG and TG. No significant differences were found between the CG and rCG and rTG compared to the TG.
Gumus et al. (2014) [30]	Internal connection	*n* = 40 CG (*n* = 10): No coating G1 (*n* = 10): CHX G2 (*n* = 10): Saliva G3 (*n* = 10): Blood	Titanium Grade IV	25 N cm	CHX Mouthwash 0.3% diluted to 0.1% Human saliva Blood (fresh)	RTV after thermocycling	Thermocycling: Cycles: 1500 Temperature: 5–55 °C Dwell time: 60 s	Mean RTV: CG: 21 N cm G1: 20.28 N cm G2: 19.3 N cm G3: 18.9 N cm With significant difference between CG and G3.
Koosha et al. (2020) [31]	Internal connection	*n* = 45 CG (*n* = 9): No coating G1 (*n* = 9): CHX G2 (*n* = 9): Saliva G3 (*n* = 9): Blood G4 (*n* = 9): Fluoride	NR	25 N cm	CHX Mouthwash 0.2% Human saliva Blood (fresh) Fluoride Mouthwash 0.2%	RTV after Thermocycling	Thermocycling: Cycles: 1500 Temperature: 5–55 °C Dwell time 60 s	Mean RTV: CG: 16.5 N cm G1: 19.74 N cm G2: 13.65 N cm G3: 16.3 N cm G4: 15.92 N cm The highest RTV was in G1, which was significantly higher than the CG. The greatest decrease in RTV was in G2 with a significant difference to CG. No significant difference was found for G3 or G4 compared with the CG.
Lyra et al. (2023) [32]	External connection and internal with conical connection	*n* = 60 Divided into 2 groups: EC (*n* = 30) and CC (*n* = 30). Each group was divided according to the coating: CG (*n* = 10): No coating G1 (*n* = 10): Adhesive medium strength G2 (*n* = 10): Adhesive high strength	Ti-6Al-4V alloy	32 N cm for the EC and 20 N cm for CC	Loctite^®^ 277 Loctite^®^ 242	‰ Torque loss after cyclic loading	Cyclic loading: Cycles: 1,200,000 Load: 133 N Frequency: 1.3 Hz	% torque loss in EC Group: CG: 27.5%, G1: 11.9% G2: 5.9% % torque loss in CC Group: CG: 4.5% G1: 1% G2: −44.5% Both groups (EC and CC) with significant difference between CG and the others, but not between G1 and G2. Although, the mean RTV on G2 in CC samples were higher than the installation torque (28.9 N cm)
Mostafavi et al. (2021) [33]	Internal with conical connection	n = 50 CG (*n* = 10): No coating G1 (*n* = 10): Blood G2 (*n* = 10): Saliva G3 (*n* = 10): CHX G4 (*n* = 10): Fluoride	Titanium Grade IV	30 N cm	Blood (fresh) Saliva (Human) CHX Mouthwash 0.2% Fluoride Mouthwash 0.2%	RTV after cyclic loading	Thermocycling: Cycles: 1500 Temperature: 5–55 °C Dwell time 60 s Cyclic loading Cycles: 500,000 Load: 100 N Frequency: 1 Hz	Mean RTV: CG: 19 N cm G1: 21.1 N cm G2: 20.56 N cm G3: 22.89 N cm G4: 24 N cm All specimens, except G2, displayed significantly higher RTV when compared to the CG.
Nigro et al. (2010) [34]	External connection	*n* = 20 CG (*n* = 10): No coating TG (*n* = 10): Saliva	TorqTite^®^	32 N cm	Artificial saliva	RTV	NP	Mean RTV: CG: 27.5 N cm TG: 31.5 N cm With significant differences.
Ozdiler et al. (2021) [35]	Internal with conical connection	*n* = 84 4 different groups A, B, C and D (*n* = 21 × 4) with 4 different implant taper angles CG (*n* = 7): No coating G1 (*n* = 7): CHX G2 (*n* = 7): Silicone gel	NR	NR	CHX gel 2% Silicone gel (KieroSeal^®^)	% of torque loss after dynamic loading	Dynamic loading cycles: 500,000 Load: 50 N Frequency: 1 Hz The stylus motion started 2 mm away from the center of the full metal crown, moved 3 mm vertically and slid 2 mm in the horizontal plane toward the center at each cycle.	Mean % torque loss in Group A: CG: 6.29% G1: 7.71% G2: 15.52% Mean % torque loss in Group B: CG: 8.76% G1: 10.62% G2: 16.14% Mean % torque loss in Group C: CG: 8.81% G1: 10.75% G2: 17.33% Mean % torque loss in Group D: CG: 12.29% G1: 13.57% G2: 19.95% With significant difference only for G2 in all groups (A, B, C and D).
Ryu et al. (2020) [36]	External connection	*n* = 20 CG (*n* = 10): No coating TG (*n* = 10): Adhesive	Titanium	30 N cm	Loctite^®^ 243	RTV	NP	Mean RTV: CG: 20.31 N cm TG: 32.4 N cm With significant difference
Sam et al. (2020) [37]	Nobel analogs, connection NR	*n* = 40 CG (*n* = 10): No coating G1 (*n* = 10): Saliva G2 (*n* = 10): Blood G3 (*n* = 10): Normal saline	NR	20 N cm	Artificial saliva Blood (origin not reported) Normal saline	RTV	NP	Mean RTV: CG: 19.5 N cm G1: 17.5 N cm G2: 31.5 N cm G3: 31 N cm With significant difference for CG and G2 and G3.
Seloto et al. (2018) [23]	External connection	*n* = 40 CG (*n* = 10): No coating G1 (*n* = 10): Anaerobic sealing agent for medium torque G2 (*n* = 10): Sealing agent for high torque G3 (*n* = 10): Cyanoacrylate-based bond	NR	32 N cm	Anaerobic sealing agent for medium torque (ASMT) Anaerobic agent for high torque (ASHT) Cyanoacrylate-based bond (CYAB)	Screw Preload RTV	NP	Mean screw preload: GC: 32.1 N cm G1: 32.1 N cm G2: 32.1 N cm G3: 32.3 N cm Mean RTV: GC: 24.6 N cm G1: 24.3 N cm G2: 51.0 N cm G3: 47.7 N cm Comparing the groups, similar RTV values were found in CG and G1 and both were significantly lower than the RTV in G2 and G3. However, no significant difference was found between G2 and G3.
Seloto et al. (2020) [38]	External connection	*n* = 24 CG (*n* = 12): No coating TG (*n* = 12): Anaerobic sealing gel	NR	30 N cm	Anaerobic sealing gel (Loctite 2400)	Screw preload RTV after cyclic loading	Cyclic loading: Cycles: 1,000,000 Load: 130 N Frequency: 2 Hz Sample angulation: 30° off-axis	Mean screw preload values: CG: 30.13 N cm TG: 30.12 N cm Mean RTV: CG: 24.26 N cm TG: 38.29 N cm With significant difference for the RTV.
Shemtov et al. (2023) [39]	Internal Connection	*n* = 43 CG (*n* = 8): No coating G1 (*n* = 7): Saliva G2 (*n* = 7): CHX Mouthwash G3 (*n* = 7): CHX gel G4 (*n* = 7): Blood G5 (*n* = 7): Fluoride	NR	30 N cm	Saliva (human) CHX Mouthwash 0.2% CHX gel 2% Blood (fresh) Fluoride Mouthwash 0.025%	RTV1: RTV before cyclic loading in 2 samples of each group RTV2: RTV after cyclic loading, 6 samples in CG, and 5 samples in each group (G1, G2, G3, G4 and G5)	Cyclic loading: Cycles: 360,000 Load: 0–200 N Frequency: NR Sample angulation: 30° off-axis	Mean RTV1: CG: 26.81 N cm G1: 26.88 N cm G2: 25.73 N cm G3: 26.89 N cm G4: 25.5 N cm G5: 26.61 N cm No significant differences were found Mean RTV2: CG: 36.8 N cm G1: 29.71 N cm G2: 23.56 N cm G3: 24.79 N cm G4: 29.71 N cm G5: 17.42 N cm No significant difference was observed between the CG and G1 and G4, but all of the 3 groups statistically differed from G2, G3 and G5. No significant difference was observed between G2, G3 and G5.
Sun et al. (2022) [40]	Internal with conical connection	*n* = 45 CG (*n* = 15): No coating G1 (*n* = 15): Saliva G2 (*n* = 15): Mouthwash	NR	32 N cm	Artificial saliva mouthwash (Listerine^®^) (ingredients NR)	RTV before cyclic loading (RTV1)—only with 5 samples RTV after cyclic loading They registered RTV2 after the first load cycle, the liquid was added again and the second cycle was performed, after which RTV3 was registered	Cyclic loading Cycles: First cycle 300,000 and second cycle 600,000 Load: 20–200 N Frequency: 15 Hz	Mean RTV1: CG: 22.82 N cm G1: 24.16 N cm G2: 25.52 N cm With significant difference between G2 and CG. Mean RTV2: the authors did not show the specific results but found significant differences between G1 and CG, G2 and CG and G1 and G2. The higher RTV was in G2, then in G1 and then in CG. RTV3: the authors did not show the specific results but found significant differences between G2 and CG and G2 and G1. The higher RTV was found in G2, then in G1 and, lastly, in CG.
Yang et al. (2022) [41]	Internal with conical connection	*n* = 484 Divided in 11 groups (*n* = 44 × 11) CG: No coating G1: Blood G2: Saliva G3: Fluoride G4: CHX G5: Blood and saliva G6: Blood and fluoride G7: Blood and CHX G8: Saliva and Fluoride G9: Saliva and CHX G10: Fluoride and CHX	Titanium Grade IV	30 N cm	Blood (fresh) Saliva (human) Fluoride gel 0.2% CHX Mouthwash 0.2%	RTV after cyclic loading	Thermocycling: Cycles: 1500 Temperature: 5–55 °C Dwell time 60 s Cyclic loading Cycles: 500,000 Load: 100 N Frequency: 1 Hz	Mean RTV: CG: 18 N cm G1: 22.12 N cm G2: 21.56 N cm G3: 24 N cm G4: 21.89 N cm G5: 22 N cm G6: 20.11 N cm G7: 22.56 N cm G8: 23.51 N cm G9: 23.89 N cm G10: 21.02 N cm The Tukey test showed that CG and coating groups, except saliva, differed significantly. This test also showed that CG and combination groups differ significantly. The test also showed that the blood group and fluoride group, the saliva group and CHX group and the saliva group and fluoride group differed significantly.
Yu et al. (2020) [22]	Internal connection and internal with conical connection	*n* = 30 3 different implant systems: Nobel^®^ (IC), Straumann^®^ (ICC) and Wego^®^ (ICC) (*n* = 10 × 3) Each system had 2 groups: CG (*n* = 5): No coating TG (*n* = 5): Silicone gel	NR	20 N cm for Wego^®^ 35 N cm for Straumann^®^ ICC and Nobel^®^ IC	Silicone gel (GapSeal^®^)	% torque loss before and after cyclic loading	Cyclic loading Cycles: 345,600 Load: 20–200 N; Frequency: 2 Hz; Sample angulation: 30° off-axis	Mean value for initial torque loss for Nobel^®^: CG: 24% TG: 17.71% Mean value for initial torque loss for Straumann^®^: CG: 17.71% TG: 10.86% Mean value for initial torque loss for Wego^®^: CG: 22% TG: 15% Mean value for final torque loss for Nobel^®^: CG: 74.86% TG: 58.86% Mean value for final torque loss for Straumann^®^: CG: 39.43% TestG: 25.14% Mean value for final torque loss for Wego^®^ CG: 71% TG: 45% The results showed the sealing gel used significantly decreased the initial and the final torque loss for all the 3 systems.

**Table 2:** Risk of bias.

	Arshad et al. [26]	Asli et al. [27]	Basilio et al. [28]	Biscoping et al. [29]	Felix et al. [7]	Gumus et al. [30]	Koosha et al. [31]	Lyra et al. [32]	Mostafavi et al. [33]	Nigro et al. [34]	Ozdiler et al. [35]	Ryu et al. [36]	Sam et al. [37]	Seloto et al. [23]	Seloto et al. [38]	Shemtov et al. [39]	Sun et al. [40]	Yang et al. [41]	Yu et al. [22]
Sample size calculation	0	2	2	1	0	2	2	2	0	2	2	2	0	1	2	2	2	2	2
Adequate statistical analysis	0	0	0	1	0	0	0	0	0	0	0	0	0	0	0	0	0	0	0
Appropriate control group	0	0	0	0	0	0	0	0	0	0	0	0	0	0	0	0	0	0	0
Tests conducted according to a standardized protocol	2	2	0	2	0	2	2	2	1	2	2	2	2	2	0	0	0	1	0
Test substance defined	0	0	0	0	0	0	0	0	0	0	0	0	0	0	0	0	1	0	0
Study characteristic description	1	1	0	1	0	0	1	0	0	0	1	0	1	1	1	1	1	0	1
Blinding of researchers	0	2	2	2	1	2	2	2	2	2	2	2	2	2	2	2	2	2	2
Experiment realized by a single operator	0	2	2	2	1	0	2	0	2	2	1	2	2	2	0	2	2	2	2
Statement of conflict of interest/funding was registered	0	0	0	0	0	0	2	0	2	2	0	2	0	0	0	0	0	0	2
Authors accounted for confounding variables	0	0	0	1	0	0	0	0	1	2	0	0	2	0	0	0	0	0	0
Incomplete outcome data	0	0	0	1	0	0	0	0	0	0	0	0	0	0	0	0	0	0	0
Selective outcome reporting	0	0	1	1	0	0	0	0	0	0	1	0	0	0	0	0	1	0	0
Total	3	9	7	12	2	6	11	6	8	12	9	10	9	8	5	7	9	7	9

## Data Availability

The data that support the findings of this study are available from the corresponding author upon request.

## Supplementary Materials

The following supporting information can be downloaded at: https://www.mdpi.com/article/10.3390/ma17205053/s1, Table S1: Prisma Checklist.
